# Randomized, double-blind, four-arm pilot study on the effects of chicken essence and type II collagen hydrolysate on joint, bone, and muscle functions

**DOI:** 10.1186/s12937-023-00837-w

**Published:** 2023-03-15

**Authors:** Chun-Chieh Chen, Shih-Sheng Chang, Chih-Hsiang Chang, Chih-Chien Hu, Yoshihiro Nakao, Shan May Yong, Yen Ling Ow Mandy, Chia Juan Lim, Eric Kian-Shiun Shim, Hsin-Nung Shih

**Affiliations:** 1 Department of Orthopedic Surgery, Chang Gung Memorial Hospital, Linkou, No. 5, Fuxing Street, Guishan District, Taoyuan City 333, Taiwan; 2 College of Medicine, Chang Gung University, No. 259, Wenhua 1st Road, Guishan District, Taoyuan City, 333 Taiwan; 3 Bone and Joint Research Center, Chang Gung Memorial Hospital, Linkou, No. 5, Fuxing Street, Guishan District, Taoyuan City, 333 Taiwan; 4 Graduate Institute of Clinical Medical Sciences, College of Medicine, Chang Gung University, No. 259, Wenhua 1St Road, Guishan District, Taoyuan City, 333 Taiwan; 5 Research and Development, Suntory Beverage and Food Asia, 3 Biopolis Drive, #06-14/19, Synapse, Singapore, 138623 Singapore

**Keywords:** Knee osteoarthritis, Chicken essence, Collagen hydrolysate, Nutritional supplements, Joint pain

## Abstract

**Background:**

Knee osteoarthritis (OA) is a leading cause of disability among older adults. Medical and surgical treatments are costly and associated with side effects. A natural nutraceutical, collagen hydrolysate, has received considerable attention due to its relieving effects on OA-associated symptoms. This study investigated the effects of hydrolyzed collagen type II (HC-II) and essence of chicken (BRAND'S Essence of Chicken) with added HC-II (EC-HC-II) on joint, muscle, and bone functions among older adults with OA.

**Methods:**

Patients (*n* = 160) with grade 1–3 knee OA according to the Kellgren–Lawrence classification system, joint pain for ≥ 3 months, and a Western Ontario and McMaster Universities Osteoarthritis Index (WOMAC) score of > 6 were randomly assigned with equal probability to consume EC-HC-II, HC-II, glucosamine HCl, or a placebo for 24 weeks in combination with resistance training. Outcome measurements were WOMAC score, visual analogue scale (VAS) pain score, grip strength, fat-free mass (FFM), and bone mass.

**Results:**

All groups exhibited similar levels of improvement in WOMAC index scores after 24 weeks. HC-II significantly reduced VAS pain score by 0.9 ± 1.89 (*p* = 0.034) after 14 days. A repeated-measures analysis of variance showed that HC-II reduced pain levels more than the placebo did (mean ± standard error: − 1.3 ± 0.45, *p* = 0.021) after 14 days; the EC-HC-II group also had significantly higher FFM than the glucosamine HCl (*p* = 0.02) and placebo (*p* = 0.017) groups and significantly higher grip strength than the glucosamine HCl group (*p* = 0.002) at 24 weeks.

**Conclusion:**

HC-II reduces pain, and EC-HC-II may improve FFM and muscle strength. This suggests that EC-HC-II may be a novel holistic solution for mobility by improving joint, muscle, and bone health among older adults. Large-scale studies should be conducted to validate these findings.

**Trial registration:**

This trial was retrospectively registered at ClinicalTrials.gov (NCT04483024).

**Supplementary Information:**

The online version contains supplementary material available at 10.1186/s12937-023-00837-w.

## Background

Knee osteoarthritis (OA) is a disease affecting an entire joint, including the articular cartilage, subchondral bone, synovial tissues, and menisci [1], and is a leading cause of disability among older adults [2]. The World Health Organization Global Burden of Disease Study conducted in 21 epidemiological regions worldwide reported a 26.6% increase in the burden of knee OA between 1990 and 2010 [3].

Therapies for OA include over-the-counter analgesics, nonsteroidal anti-inflammatory drugs, intra-articular injections of corticosteroids or hyaluronic acid (HA), tramadol, and other opioid analgesics [4, 5]. Although these therapies alleviate short-term symptoms, their overall effect on OA pathophysiological progression is limited [6], and total joint replacement is the only long-term solution for OA.

Individuals with OA typically switch to natural nutraceuticals for pain and discomfort relief. Nutraceuticals or their components are functional foods and natural products that have medicinal, therapeutic, or other health benefits. Glucosamine HCl and chondroitin are the most commonly used nutraceuticals that alleviate pain associated with arthritis [7]. Collagen hydrolysate (CH) has also received considerable attention due to its ability to relieve OA-associated symptoms [8, 9]. Because articular cartilage collagen fibrils mostly comprise type II collagen with other minor collagens [10], CH may reduce OA-associated symptoms by providing supporting collagen fibrillar network development, which promotes tensile strength in the articular cartilage matrix [11, 12]. Studies have reported the beneficial effects of CH on joint health [13, 14] and pain relief [11, 12, 15, 16].

Considering these findings, we developed hydrolyzed collagen type II (HC-II), a type II CH derived from chicken cartilage, and BRAND'S Essence of Chicken (EC) plus type II hydrolyzed collagen HC-II (EC-HC-II), a chicken essence supplement with additional HC-II. HC-II is a naturally occurring soluble matrix of hydrolyzed collagen type II, chondroitin sulfate, and HA. Its composition is similar to that of the human articular cartilage lining in the synovial joints. This randomized, double-blind, four-arm pilot study was undertaken to investigate the effects of EC-HC-II on joint, bone, and muscle functions in patients with OA and its tolerability.

## Methods

### Study design

This randomized, double-blind, four-arm, pilot study evaluated the effects of EC-HC-II on joint, bone, and muscle functions in patients with knee OA (NCT04483024). After enrollment, participants were randomly allocated in equal proportions to four supplement groups: EC-HC-II, HC-II, glucosamine HCl, and placebo. Eligible participants were randomly assigned to groups at a 1:1:1:1 ratio. A block randomization list with a block size of eight was generated using Statistical Analysis Software (SAS). Participants were instructed to consume one bottle (68 mL) of the experimental product daily in the morning (after breakfast) for 24 weeks and perform resistance exercises for 30 min twice per week according to training manual. Product consumption and resistance training were recorded on participant diary cards. Each participant undertook follow-up assessments at 8, 16, and 24 weeks. This study designed and administered a food questionnaire on the participant’s calcium, vitamin D, and protein intake every week before assessments. Throughout the study, the use of analgesics as rescue medication was permitted.

The study was approved by the Institutional Review Board of blinded information and conducted in accordance with the Declaration of Helsinki and local regulations. Written informed consent was obtained from participants before beginning the study.

### Participants

The study enrolled participants aged 45–75 years that had a body mass index within the healthy range (18.0–30.0 kg/m^2^), weighed at least 40 kg, had experienced knee pain for ≥ 3 months, had a Western Ontario and McMaster Universities Osteoarthritis Index (WOMAC) total pain score of ≥ 6, presented with mild to moderate knee OA (grade 1–3) according to the Kellgren–Lawrence classification system, had had a loss in muscular strength or physical performance for > 1 year, and were willing to discontinue hormone therapy and dietary supplements during the study period.

Individuals with active viral or bacterial infection based on clinical examinations, a history of rheumatoid or other types of arthritis, renal dysfunction, psychiatric disorders, diabetes, stroke or myocardial infarction, intellectual disability, schizophrenia, gout, Paget’s disease of bone or spinal disc herniation, expected knee arthroscopy or arthroplasty, or life-threatening pathology were excluded. Patients that had, at the time of enrollment, received treatments including antiosteoporotic therapy within the last year, intra-articular injection applied at the target knee joint (most painful knee during screening) within the last 3 months (6 months for HA), or corticosteroids were excluded. The study also excluded patients who were intolerant to protein-based food or supplements, pregnant or lactating women, and patients with alcohol abuse or addiction.

### Experimental products

The study investigated two experimental products, HC-II and EC-HC-II, in comparison with glucosamine HCl and a placebo. Both experimental products and comparators were provided in 68-mL bottles by Suntory Beverage and Food Asia (Changhua Taiwan, Good Hygiene Practice certified). HC-II doses contained 2.0 g HC-II derived from chicken sternal cartilage by enzymatic hydrolysis with a molecular weight of less than 10,000 Dalton, providing a naturally occurring composite of CH (66.5%), depolymerized chondroitin sulfate (18%), and HA (11%). The uncharacterized components of the sternal cartilage accounted for the remaining 4.5%. EC-HC-II doses contained 2.0 g of HC-II collagen and 5.81 g of EC with proteins and peptides. EC was produced using a water extraction process from chicken meat for several hours under high temperature, followed by centrifugation to remove fat and cholesterol, vacuum concentration, and sterilization at high temperature and pressure before bottling. The protein and amino acid content of HC-II and EC-HC-II are shown in Table 1. Each dose of the active comparator contained 1.5 g glucosamine HCl. The placebo formulation included 6.8 g maltodextrin, 0.007 g xanthan gum, and 0.43 g yeast extract. Xanthan gum was used to replicate the texture of EC and EC-HC-II, and yeast extract was used to mimic the flavor of EC and EC-HC-II. All products in the glass bottles were isocaloric, identical in appearance, and had similar flavors and textures.

**Table 1:** Composition of EC-HC-II and HC-II (analyzed using AOAC Official Methods of Analysis 994.12)

**Parameters**	**EC-HC-II (%)**	**HC-II (%)**
Protein	10.16	1.956
Total amino acids	8.212	1.702
Alanine	0.631	0.123
Arginine	0.588	0.137
Aspartic acid	0.575	0.115
Cystine	0.026	0.013
Glutamic acid	1.282	0.235
Glycine	1.157	0.203
Histidine	0.121	0.055
Hydroxyproline	8.212	0.129
Isoleucine	0.198	0.049
Leucine	0.467	0.098
Lysine	0.372	0.083
Methionine	0.075	0.027
Phenylalanine	0.317	0.048
Proline	0.651	0.157
Serine	0.263	0.054
Taurine	0.055	0.007
Threonine	0.241	0.057
Tryptophan	0.032	0.007
Tyrosine	0.131	0.027
Valine	0.246	0.076
Minerals (sodium, calcium, potassium, chloride)	0.721	0.188
Total fat	0.04	0.009

*HC-II* hydrolyzed collagen type II, *EC-HC-II* essence of chicken with HC-II

### Assessments of joint health

WOMAC and visual analogue scale (VAS) pain scores were used to assess joint health. Data on the WOMAC score were gathered at 8, 16, and 24 weeks, whereas those on the VAS pain score were gathered at 7 and 14 days. The WOMAC is a widely used, proprietary, standardized questionnaire for evaluation of OA severity, including pain, stiffness, and physical function of the joints. Higher WOMAC scores indicate increased pain, stiffness, and functional limitations [17]. The VAS pain score was measured using patient self-assessment. The VAS is a 100-mm scale used to record the intensity of patients’ most severe pain; a higher VAS score indicates greater pain.

### Assessments of muscle strength

Grip strength and fat-free mass (FFM) measurements were used to assess muscle strength. Grip strength was measured three times each using a DynamoMeter (Smedley, TTM; Tokyo, Japan) at 8, 16, and 24 weeks, with the maximum value recorded. FFM was calculated at 24 weeks as follows: FFM = body weight − fat mass (FM), where FM was measured using dual-energy X-ray absorptiometry (DXA; Hologic Horizon DXA System, United States).

### Assessments of bone health

The bone mass of the lumbar spine, left hip, and right hip was assessed using DXA at 24 weeks.

### Assessments of patient-reported outcomes

The 36-Item Short-Form Survey (SF-36) [18] was used to evaluate patient outcomes at 8, 16, and 24 weeks. The SF-36 is a patient-reported survey of patient health. It consists of eight scales (vitality, physical function, bodily pain, general health perceptions, physical role activity function, emotional role activity function, social activity function, and mental health). Each scale is scored from 0‒100, with lower scores indicating increased disability.

### Statistical analysis

The sample size was estimated based on another study with an estimated mean of 10.26, standard deviation (SD) of 13.81, two-sided alpha level of 0.05, statistical power of 80%, and an anticipated dropout rate of 25% [9]. A total of 144 participants (36 per study group) was considered sufficient for this study.

The participants had a supplementation compliance rate of ≥ 70% and did not deviate from the study protocol in a manner that would have led to their withdrawal from the study. The Kruskal–Wallis test was used to compare continuous variables (ie, WOMAC score, VAS pain score, grip strength, FFM, and bone mass) among groups. The chi-squared test was used to compare categorical variables (eg, sex and baseline characteristics) among groups. Changes in continuous variables from baseline to the study endpoint were analyzed using paired *t*-tests and the Wilcoxon signed-rank test.

To investigate the effects of supplementation when minimal resistance training was performed, a subgroup analysis was also conducted with participants with poor resistance training compliance (average minutes spent training in the 10th percentile). Analysis of variance (ANOVA) and analysis of covariance using a repeated-measures mixed-effect model with the supplement × visits interaction included as a fixed effect factor was used to compare the difference in continuous efficacy endpoints among groups, with baseline values, sex, number of follow-up visits, and supplementation serving as covariates. Covariates were selected by identifying key physiological factors, such as differences in FFM and bone mass loss, in terms of sex and baseline values reflecting different disease severities, which may have influenced the measured outcome*.*

Statistical analyses were performed using SAS version 9.4 (SAS Institute, Cary, NC, United States) and GraphPad Prism version 5.0 (San Diego, California, United States). A *p* value < 0.05 indicated significance.

## Results

**Fig. 1:**
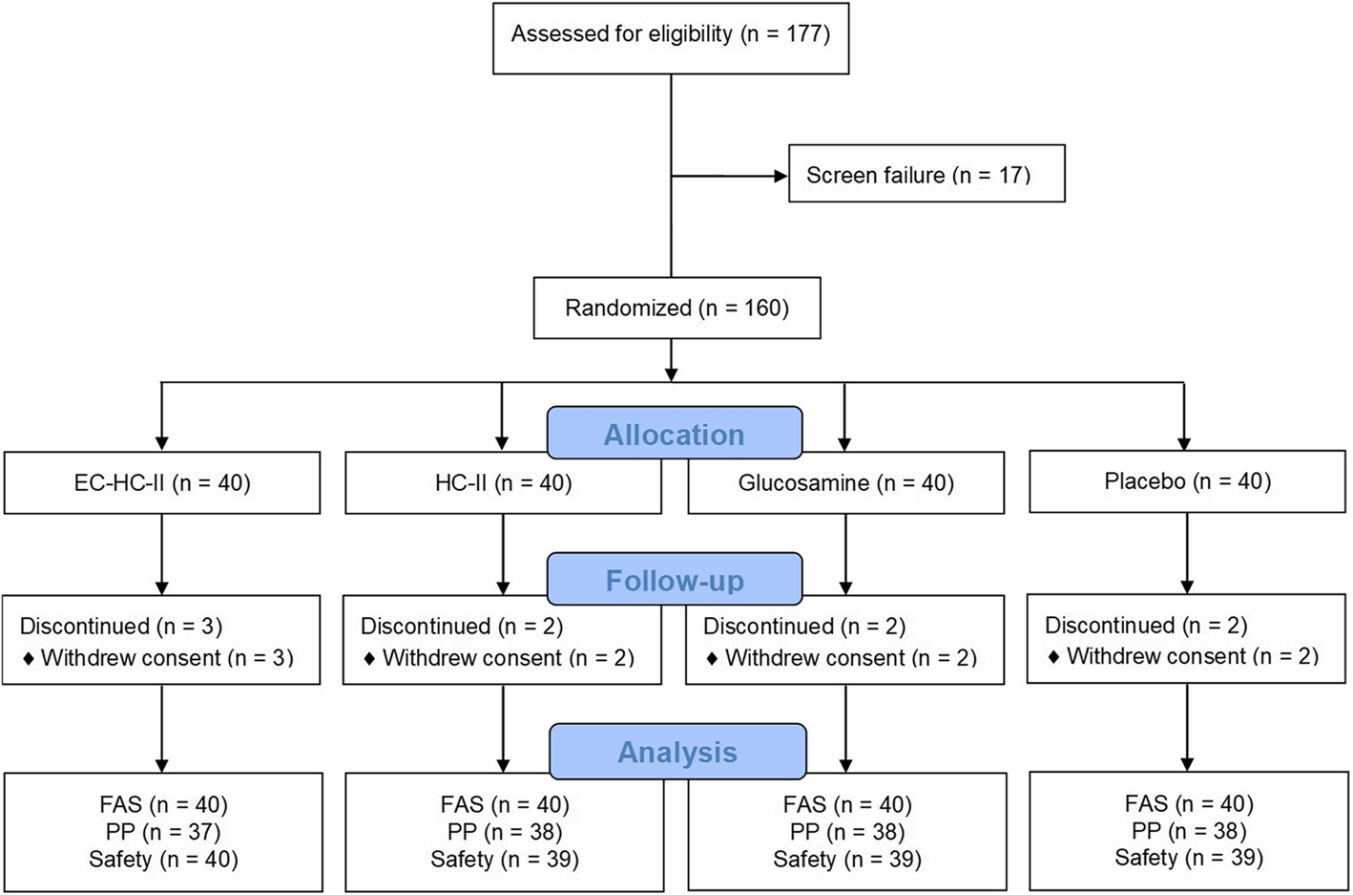
Flow of study

A total of 160 participants were recruited from November 2018 to March 2019 and allocated equally among four groups (*n* = 40 in each group). Nine participants (5.6%) withdrew from the study due to withdrawal of consent and were excluded from analysis (Fig. 1). High compliance to the prescribed resistance training regimen was achieved in all groups during the study. In each group, the mean duration of exercise was within 110-130% of the prescribed duration.

### Participant baseline characteristics

The baseline characteristics of participants are presented in Table 2. No significant difference among the groups existed at baseline. The mean ages of all groups were 56.4 to 59.1 years. Approximately 84.8% of participants were women, and most were undergoing menopause (87.9%, 73.3%, 75.0%, and 60.6% in the EC-HC-II, HC-II, glucosamine HCl, and placebo groups, respectively). Almost all participants did not smoke. The groups did not significantly differ in baseline knee OA severity existed and over half of participants presented with Kellgren–Lawrence grade 1 OA.

**Table 2:** Baseline characteristics of participants

**Parameters**	**EC-HC-II** ***n* = 37**	**HC-II** ***n* = 38**	**Glucosamine HCl** ***n* = 38**	**Placebo** ***n* = 38**	***p* value**
Age, mean (SD; years)	57.3 (7.14)	59.8 (7.66)	59.1 (8.22)	56.4 (6.42)	0.240
Female, n (%)	33 (89.2)	30 (78.9)	32 (84.2)	33 (86.8)	0.638
Height, mean (SD; cm)	156.9 (6.36)	155.8 (7.78)	157.0 (7.34)	158.5 (6.34)	0.480
BMI, mean (SD; kg/m^2^)	23.9 (3.49)	24.4 (3.26)	24.6 (2.6)	24.4 (3.34)	0.738
Weight, mean (SD; kg)	58.5 (8.57)	59.4 (9.69)	61.0 (8.72)	61.2 (10.14)	0.533
Menopause, n (% of women)	29/33 (87.9)	22/30 (73.3)	24/32 (75.0)	20/33 (60.6)	0.258
Smoking habit, n (%)	2 (5.4)	0 (0.0)	0 (0.0)	3 (7.9)	0.276
Alcohol addiction, n (%)	0 (0.0)	0 (0.0)	0 (0.0)	0 (0.0)	NA
Knee OA grade according to Kellgren–Lawrence classification system, n (%)					
1	30 (81.1)	25 (65.8)	22 (57.9)	26 (68.4)	0.299
2	4 (10.8)	10 (26.3)	13 (34.2)	11 (28.9)	
3	3 (8.1)	3 (7.9)	3 (7.9)	1 (2.6)	

Kruskal–Wallis test and chi-square test were applied for continuous and categorized variables, respectively

*HC-II* hydrolyzed collagen type II, *EC-HC-II* essence of chicken with HC-II, *SD* standard deviation, *OA* osteoarthritis, *NA* not available

### Effects on joint health

**Fig. 2:**
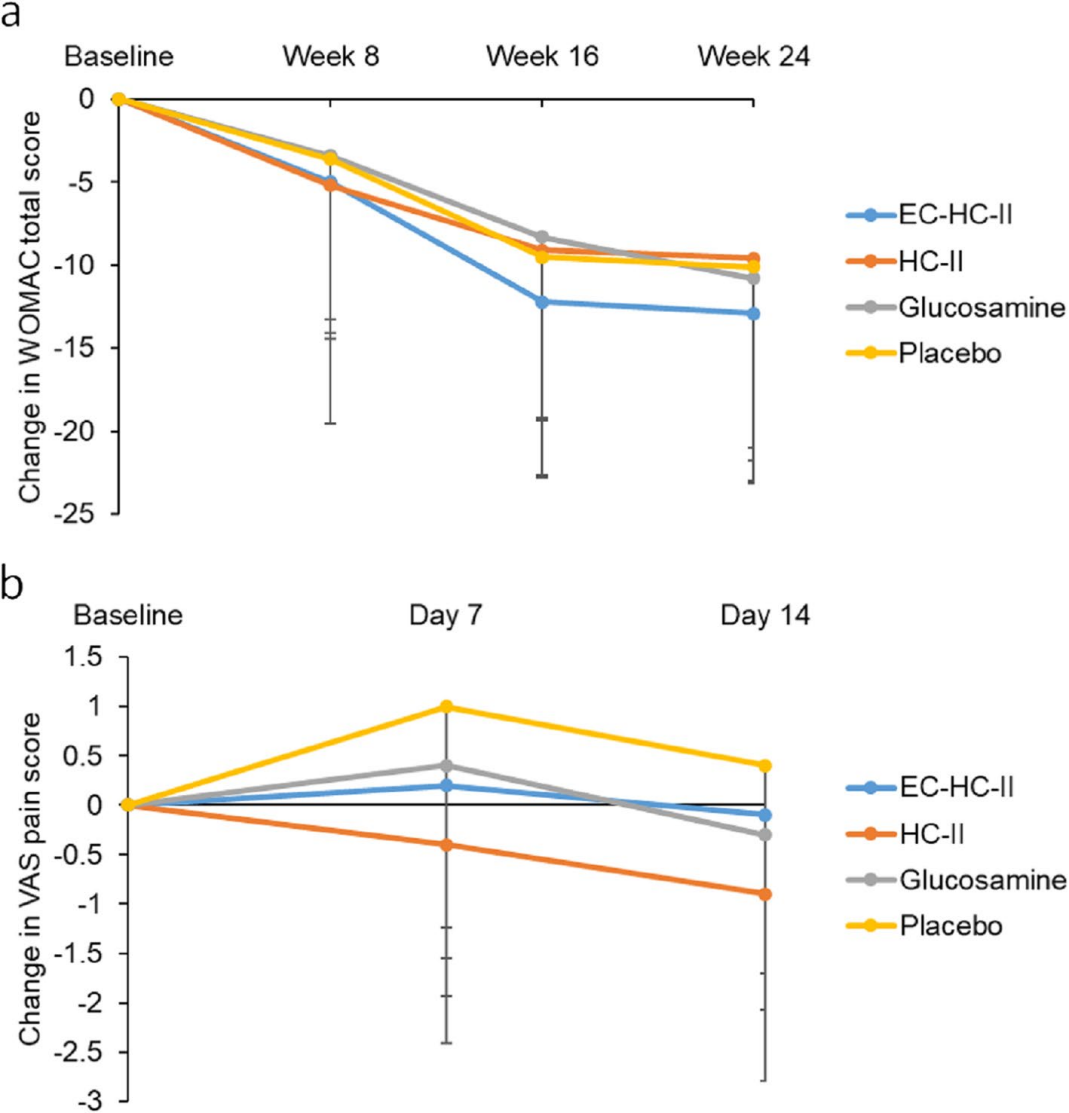
Mean change in outcomes at visits. **a** Total WOMAC score; **b** VAS pain score. Abbreviations: EC, Essence of Chicken; WOMAC, Western Ontario and McMaster Universities Osteoarthritis Index; VAS, visual analogue scale

WOMAC total score significantly decreased in all groups after 24 weeks (Fig. 2a). However, the groups did not significantly differ in WOMAC total score after 24 weeks (*p* = 0.848). VAS pain score on day 14 significantly differed among groups (*p* = 0.039; Fig. 2b). The HC-II group had a significant reduction (0.9 ± 1.89; *p* = 0.034) in VAS pain score after 14 days. By contrast, in the placebo group, VAS pain score significantly increased by 1.0 ± 2.24 from baseline to day 7 (*p* = 0.012). Repeated-measures ANOVA adjusted for the interaction of supplement × visits indicated that the HC-II group had a greater decrease in pain than the placebo group (*p* = 0.021; Table 3).

**Table 3:** Repeated-measures ANOVA of VAS pain score on day 14

**Factors**	**LSM (SE)**^a^	**95% CI**	***p* value**^**‡**^
EC-HC-II vs. HC-II	0.7 (0.45)	(− 0.51, 1.89)	0.748
EC-HC-II vs. glucosamine HCl	− 0.0 (0.45)	(− 1.21, 1.18)	> 0.999
EC-HC-II vs. placebo	− 0.6 (0.45)	(− 1.83, 0.57)	0.983
HC-II vs. glucosamine HCl	− 0.7 (0.45)	(− 1.90, 0.48)	0.687
HC-II vs. placebo	− 1.3 (0.45)	(− 2.51, − 0.13)	0.021*
Glucosamine HCl vs. placebo	− 0.6 (0.45)	(− 1.80, 0.58)	> 0.999

*LSM* least squares mean, *SE* standard error, *CI* confidence interval, *HC-II* hydrolyzed collagen type II, *EC-HC-II* essence of chicken with HC-II, *VAS* visual analogue scale, *ANOVA* analysis of variance

^*^Statistical significance

^‡^Statistical method: repeated-measures ANOVA

^a^Difference in LSM between groups

### Effects on muscle strength

Changes in FFM and grip strength after 24 weeks did not significantly differ among groups (data not shown). Initial ANOVA in the adjusted analyses revealed that supplementation and the interaction of supplementation × sex had significant effects on FFM and grip strength. ANOVA adjusted for these significant factors demonstrated significantly increased FFM after 24 weeks in the EC-HC-II group compared with in the glucosamine HCl (*p* = 0.02) and placebo (*p* = 0.017) groups (Supplementary Table 1). Similarly, the EC-HC-II group had significantly greater grip strength than the glucosamine HCl group at 24 weeks (*p* = 0.002).

### Effects on bone mass

After 24 weeks of supplementation, all participants presented with consistent bone mass in the lumbar spine, left hip, and right hip. The groups did not significantly differ in bone mass after 24 weeks of supplementation (data not shown).

### Subgroup analysis of poor training compliance

Resistance training is useful for people with OA because it can strengthen the muscles surrounding affected joints [19]. Therefore, a subgroup analysis where participants were divided by whether they had minimal compliance with resistance training (Supplementary Text and Supplementary Table 2). Results suggested that the benefits of EC-HC-II in pain relief, bone health, and muscular strength were sustained even with minimal resistance training.

### Effects on patient-reported outcomes

Participants in all four groups reported improved physical component scores in the SF-36. Moreover, the HC-II and placebo groups exhibited significant improvement in mental component scores after 24 weeks. However, no significant differences were observed among groups (Supplementary Table 3).

### Safety

EC-HC-II and HC-II were considered safe and tolerable, and no clinically significant abnormalities or changes related to EC-HC-II and HC-II were identified. The results of laboratory tests, including alanine aminotransferase, aspartate aminotransferase, total bilirubin, albumin, and total protein tests, remained normal and stable across 24 weeks.

## Discussion

This pilot, randomized, placebo-controlled study was the first clinical trial to evaluate the efficacy of HC-II, a newly developed chicken-derived CH, alone and in combination with chicken essence, a food supplement that has been consumed in Asia for a long time. The main finding was that HC-II had a fast-acting effect on reducing pain after 14 days, as measured using the VAS. Synovial inflammation is associated with joint pain in patients with OA [20]. Pro-inflammatory cytokines, including interleukins (ILs), such as IL-1 β, IL-6, and IL-8, monocyte-chemoattractant-1 (MCP-1), chemokine ligand 5 (regulated upon activation, normal T cell expressed and presumably secreted [RANTES]), and macrophage inflammatory protein-1 (MIP-1) [21–23] play crucial roles in pain development and destruction of cartilage and synovitis [24–26]. HC-II downregulated inflammatory markers, such as IL-6, IL-8, MCP-1 and MIP-1β, in chondrocytes induced by IL-1β (Supplementary Fig. 1). Furthermore, a group of bioactive compounds including the novel bioactive peptide, Gly-Pro-Ala-Gly-Pro, cyclic glycine-proline, cyclo-alanine-hydroxyproline, guanosine, and tryptophan contributed to the anti-inflammatory effects of HC-II (Supplementary Fig. 1).

WOMAC total score improved in all four groups after 24 weeks. However, the differences among groups was not significant. This result might be attributed to the following causes: (1) the psychological effects of supplementation and study regimen (exercise, consultations, and counseling) and placebo effects, (2) the physiological benefits of resistance training and lifestyle modifications, or (3) the patients’ characteristics. EC-HC-II differs from other supplements enriched with type II collagen in terms of ingredients (chicken essence), amount of collagen, and format (liquid). Other factors such as food, exercise, and environmental factors (eg, amount of sunlight and physical activities) may explain the different outcomes between our study and other studies of supplements enriched with type II collagen. With regard to patient characteristics, this study enrolled mostly patients with early or mild OA (Kellgren–Lawrence grade 1: > 68% of patients) who were not receiving OA treatment; therefore, they may have failed to exhibit significant improvements in symptoms after supplementation.

Previous trials investigating the role of collagen in treating OA have reported contrasting results. When compared with glucosamine HCl [27] and a placebo [28], treatment with type II collagen (10 g/day) for 13 weeks was more effective in reducing WOMAC scores in patients with knee OA. A magnetic resonance imaging study examining changes in the cartilage of patients with mild knee OA [12] indicated that collagen peptides increased proteoglycan levels in the knee cartilage after 6 months. These findings were consistent with in vitro data showing stimulation of extracellular matrix synthesis by collagen peptides [29, 30]. By contrast, no differences were observed in patients with OA when type II collagen was either added to glucosamine and chondroitin sulfate supplements or administered as a standard analgesic therapy [31, 32].

After 24 weeks, the EC-HC-II group exhibited higher FFM than the glucosamine HCl and placebo groups and higher grip strength than the glucosamine HCl group. Some plausible mechanisms of both EC and collagen peptides have been indicated in studies. An animal study demonstrated that EC improved exercise performance and endurance capacity due to its antioxidant properties and antifatigue effects [26]. Another preliminary in vitro study indicated the benefits of EC in the prevention of inflammation-induced muscle atrophy [33]. The effects of collagen peptides on muscle recovery [34] and lean body mass preservation [35] have been clinically demonstrated in studies involving older adults. This may support the hypothesis that combined chicken essence and CH contribute to muscle growth and strength, particularly in older adults.

Systematic reviews have concluded that resistance training is effective in managing the symptoms of OA by improving joint function and reducing pain [36, 37], possibly by enhancing muscle strength and rebalancing leg muscle activation patterns [38]. Therefore, to identify the effects of supplementation not associated with exercise, a subgroup analysis of patients with low exercise compliance was performed. All experimental arms (EC-HC-II, HC-II, and glucosamine HCl) had better outcomes than the placebo arm, although the sample size of each subgroup was small. Because overall resistance training compliance was high in this study (> 100% for all groups), this trial may underestimate the efficacy of supplements. Their effects might be more pronounced in the absence of exercise.

In addition, our study also showed that taking EC-HC-II resulted in greater improvement in left hip bone mass than taking a placebo or glucosamine HCl did among participants who undertook minimal resistance training. EC-HC-II contained a novel peptide, Gly-Pro-Glu-Gly-Ala-Pro-Gly-Lys-Asp, which was found to reduce bone resorption in an in vitro model measuring bone matrix collagen degradation by differentiated osteoclasts. We hypothesize that this is an underlying mechanism associated with increased bone mass (unpublished data). To our knowledge, only two clinical studies have shown that relative to calcitonin and calcium plus vitamin D, collagen peptides contribute more to the inhibition of bone collagen breakdown and loss of bone mineral density, respectively [39]. As such, this study contributes to the growing body of clinical evidence supporting the use of collagen for bone health.

This study has some limitations. First, physical activity was not measured at baseline, because participants, mostly aged 50 to 60 years, were considered unlikely to have undertaken any form of resistance exercise program prior to entering the study. Nevertheless, future studies can more comprehensively evaluate the effects of baseline activity level on study outcomes. Second, because the study was designed to use WOMAC score, the sample size may be inadequate for other endpoints and the heterogeneity of the small cohort might cause biases or variations in results. A post hoc analysis adjusted for the characteristics of participants within a more defined cohort showed that CH-containing products reduce OA-derived pain and increase muscle mass and strength. These promising results warrant further verification in a large-scale study. Third, the study period of 6 months may have been too short to detect a clinically significant change in joint and bone conditions. Thus, further long-term studies should be conducted to validate the effects of EC-HC-II and HC-II on locomotor function. Fourth, female predominance (84.8% of patients) might limit the generalizability of our results. Nevertheless, prevalence of knee OA is higher in women than men [40, 41]. OA in women is commonly more advanced and aggressive [42], leading to higher levels of pain and disability [43]. In a 15-year retrospective study using data from the Taiwanese National Health Insurance Research Database, the prevalence of OA and incidence rate of total knee replacement among women were 2.5–3 times higher than in men [44]. Fifth, our subgroup analysis was exploratory in nature and limited by a small sample size. Although it suggests the potential of the supplements in modulating joint, muscle, and bone function regardless of the amount of resistance training, the results require confirmation in larger studies.

EC-HC-II and HC-II were well-tolerated by participants, and no safety problems were detected. The incidence of musculoskeletal and connective tissue disorders was higher in the placebo group than in the supplement groups. Musculoskeletal and connective tissue disorders reflect joint, muscle, and bone health. Thus, we inferred that EC-HC-II and HC-II might have protective effects on bones, muscles, and joints. Nevertheless, further studies are necessary to validate these findings.

## Conclusion

HC-II and EC-HC-II are promising supplements. HC-II was found to reduce pain within 14 days; and EC-HC-II may improve FFM, muscle strength, and bone health. Although this pilot study was limited by sample size, it demonstrated that EC-HC-II, with a combination of naturally derived food supplements, may be a holistic solution for mobility by improving joint, muscle, and bone health among older adults. Large-scale studies are necessary to validate these findings.

## Supplementary Information

**Additional file 1.**

## Data Availability

All data generated or analyzed during this study are included in this published article.
