# Supplementary material for: Randomized, double-blind, four-arm pilot study on the effects of chicken essence and type II collagen hydrolysate on joint, bone, and muscle functions
Source: Nutr J. 2023 Mar 15;22:17. doi: 10.1186/s12937-023-00837-w (PMC10015953; doi:10.1186/s12937-023-00837-w)
Supplement: Supplementary file 1 — Additional file 1. [file 12937_2023_837_MOESM1_ESM.docx]

**Additional file**

**Supplementary Table 1** ANOVA results for grip strength and FFM at Week 24 after adjusting for supplement and interaction of supplementation × sex

|  | **FFM** | | |  | **Grip strength** | | |
| --- | --- | --- | --- | --- | --- | --- | --- |
|  | **LSM (SE)^†^, g** | **95% CI, g** | **p value^‡^** |  | **LSM (SE)^†^, kg** | **95% CI, kg** | **p value^‡^** |
| EC-HC-II vs. HC-II | 1532 (1122) | (-1470, 4534) | > 0.999 |  | 2.7 (1.19) | (-0.52, 5.82) | 0.162 |
| EC-HC-II vs. Glucosamine | 3434 (1148) | (361.2, 6507) | 0.020* |  | 4.6 (1.24) | (1.25, 7.87) | 0.002* |
| EC-HC-II vs. Placebo | 3610 (1188) | (432.0, 6788) | 0.017* |  | 0.2 (1.28) | (-3.22, 3.62) | > 0.999 |
| HC-II vs. Glucosamine | 1902 (1016) | (-816, 4620) | 0.379 |  | 1.9 (1.07) | (-0.95, 4.77) | 0.459 |
| HC-II vs. Placebo | 2078 (1060) | (-758, 4914) | 0.311 |  | -2.5 (1.12) | (-5.44, 0.54) | 0.179 |
| Glucosamine vs. Placebo | 176 (1088) | (-2735, 3087) | > 0.999 |  | -4.4 (1.17) | (-7.49, -1.22) | 0.002* |
| *Statistical significance  ^†^Difference in LSM between groups  ^‡^Statistical method: two-way ANOVA  *LSM* Least-squares mean, *SE* standard error, *CI* confidence interval, *EC* essence of chicken, *FFM* fat-free mass, *ANOVA* analysis of variance | | | | | | | |

**Supplementary Table 2** Subgroup results from repeated-measures analysis of VAS pain score, grip strength, and left hip bone mass in participants with training time in 10th percentile

|  | **VAS pain score** | | |  | **Grip strength** | | |  | **Left hip bone mass** | | |
| --- | --- | --- | --- | --- | --- | --- | --- | --- | --- | --- | --- |
|  | **LSM (SE)^†^** | **95% CI** | **p value^‡^** |  | **LSM (SE)^†^, kg** | **95% CI, kg** | **p value^‡^** |  | **LSM (SE)^†^**  **(T-score)** | **95% CI**  **(T-score)** | **p value^‡^** |
| EC-HC-II vs. HC-II | 0.7 (0.53) | (-1.90, 3.21) | > 0.999 |  | -- | -- | -- |  | -- | -- | -- |
| EC-HC-II vs. Glucosamine | 1.8 (0.53) | (-0.71, 4.40) | 0.148 |  | 3.9 (0.36) | (1.16, 6.60) | 0.025* |  | 0.2 (0.04) | (0.00, 0.40) | 0.049* |
| EC-HC-II vs. Placebo | -2.6 (0.53) | (-5.16, -0.05) | 0.047* |  | 0.8 (0.33) | (-1.73, 3.38) | 0.395 |  | 0.4 (0.04) | (0.15, 0.55) | 0.010* |
| HC-II vs. Glucosamine | 1.2 (0.53) | (-1.36, 3.75) | 0.512 |  | -- | -- | -- |  | -- | -- | -- |
| HC-II vs. Placebo | -3.3 (0.53) | (-5.81, -0.70) | 0.021* |  | -- | -- | -- |  | -- | -- | -- |
| Glucosamine vs. Placebo | -4.5 (0.53) | (-7.01, -1.90) | 0.006* |  | -3.1 (0.29) | (-5.26, -0.84) | 0.026* |  | 0.2 (0.04) | (-0.05, 0.35) | 0.105 |
| *Statistical significance  ^†^Difference in LSM between groups  ^‡^Statistical methods: VAS pain score and left hip bone mass: repeated-measures ANOVA; grip strength: repeated-measures mixed model  Adjusted significant factors: VAS pain score: supplement; hand grip strength: supplement and sex; left hip bone mass: supplement  The HC-II group had no patients with training time in 10th percentile, thus some data were not available.  *LSM* Least squares mean, *SE* standard error, *CI* confidence interval, *EC* essence of chicken, *VAS* visual analogue scale | | | | | | | | | | | |

Supplementary text for Supplementary Table 2:

Repeated-measures ANOVA adjusted with supplement showed that the three experimental groups resulted in greater reductions in VAS pain score than the placebo group did after 14 days (EC-HC-II vs. placebo, p = 0.047; HC-II vs. placebo, p = 0.021; glucosamine HCl vs. placebo group, p = 0.006).

Moreover, the EC-HC-II group showed higher grip strength than the glucosamine HCl group did (p = 0.025; with supplement and sex as significant factors) and higher left hip bone mass than the glucosamine HCl and placebo groups did after 24 weeks (p = 0.049 and p = 0.01, respectively; with supplement as a significant factor).

**Supplementary Table 3** Results for SF-36

| **SF-36, mean ± SD** | **EC-CH-II**  **n = 37** | **HC-II**  **n = 38** | **Glucosamine HCl**  **n = 38** | **Placebo**  **n = 38** | **p value** |
| --- | --- | --- | --- | --- | --- |
| Physical function |  |  |  |  |  |
| Baseline | 57.4 ± 17.50 | 56.1 ± 23.43 | 62.5 ± 21.86 | 60.5 ± 18.81 | 0.514 (a) |
| Week 24 | 65.7 ± 19.83 | 67.5 ± 18.48 | 68.3 ± 17.83 | 74.5 ± 16.84 | 0.175 (k) |
| change | 8.2 ± 18.75* | 11.4 ± 20.10* | 5.8 ± 20.45 | 13.9 ± 16.53* | 0.096 (k) |
| Physical role activity function |  |  |  |  |  |
| Baseline | 54.9 ± 20.15 | 55.3 ± 19.09 | 60.0 ± 17.16 | 64.5 ± 18.56 | 0.176 (k) |
| Week 24 | 63.0 ± 20.85 | 63.0 ± 17.88 | 62.3 ± 18.41 | 67.9 ± 17.93 | 0.603 (k) |
| change | 8.1 ± 24.16* | 7.7 ± 16.01* | 2.3 ± 17.70 | 3.5 ± 19.91 | 0.468 (a) |
| Bodily pain |  |  |  |  |  |
| Baseline | 65.5 ± 14.76 | 63.5 ± 18.43 | 67.0 ± 11.11 | 67.8 ± 15.02 | 0.876 (k) |
| Week 24 | 74.2 ± 14.36 | 73.1 ± 15.42 | 74.6 ± 15.48 | 75.7 ± 15.68 | 0.797 (k) |
| change | 8.7 ± 15.52* | 9.6 ± 17.55* | 7.6 ± 15.31* | 7.9 ± 19.14* | 0.962 (k) |
| General health |  |  |  |  |  |
| Baseline | 53.5 ± 16.24 | 57.8 ± 12.01 | 59.2 ± 16.21 | 60.9 ± 14.47 | 0.319 (k) |
| Week 24 | 62.2 ± 15.30 | 64.3 ± 14.34 | 61.2 ± 18.21 | 62.2 ± 17.23 | 0.658 (k) |
| change | 8.6 ± 13.52* | 6.6 ± 12.36* | 2.0 ± 13.28 | 1.3 ± 15.67 | 0.138 (k) |
| Vitality |  |  |  |  |  |
| Baseline | 56.8 ± 14.76 | 57.9 ± 15.01 | 58.9 ± 17.23 | 56.9 ± 13.22 | 0.887 (k) |
| Week 24 | 58.4 ± 17.13 | 62.5 ± 16.31 | 60.5 ± 18.95 | 61.7 ± 12.85 | 0.809 (k) |
| change | 1.7 ± 12.90 | 4.6 ± 10.94* | 1.6 ± 16.93 | 4.8 ± 14.12* | 0.619 (a) |
| Social activity function |  |  |  |  |  |
| Baseline | 73.3 ± 15.64 | 75.3 ± 14.67 | 78.9 ± 13.97 | 75.7 ± 15.90 | 0.423 (k) |
| Week 24 | 77.4 ± 14.98 | 77.6 ± 14.58 | 78.3 ± 17.12 | 80.9 ± 14.16 | 0.687 (k) |
| change | 4.1 ± 15.88 | 2.3 ± 17.16 | -0.7 ± 16.93 | 5.3 ± 18.06 | 0.512 (k) |
| Emotional role activity function |  |  |  |  |  |
| Baseline | 64.2 ± 17.77 | 59.4 ± 22.10 | 65.8 ± 18.46 | 68.4 ± 17.45 | 0.250 (k) |
| Week 24 | 69.4 ± 18.11 | 65.6 ± 16.57 | 66.2 ± 16.43 | 71.3 ± 17.40 | 0.595 (k) |
| change | 5.2 ± 20.06 | 6.1 ± 21.02 | 0.4 ± 19.47 | 2.9 ± 21.07 | 0.431 (k) |
| Mental health |  |  |  |  |  |
| Baseline | 63.0 ± 13.92 | 65.7 ± 13.91 | 62.6 ± 17.77 | 62.5 ± 12.67 | 0.760 (a) |
| Week 24 | 65.4 ± 18.00 | 68.9 ± 14.62 | 65.0 ± 17.48 | 67.8 ± 13.34 | 0.662 (a) |
| change | 2.4 ± 11.34 | 3.3 ± 13.77 | 2.4 ± 11.07 | 5.3 ± 11.91* | 0.783 (k) |
| Physical component^1^ |  |  |  |  |  |
| Baseline | 57.8 ± 12.31 | 58.1 ± 14.93 | 62.2 ± 11.69 | 63.4 ± 13.20 | 0.155 (a) |
| Week 24 | 66.3 ± 14.83 | 67.0 ± 13.30 | 66.6 ± 13.57 | 70.1 ± 14.29 | 0.539 (k) |
| change | 8.4 ± 13.25* | 8.9 ± 11.57* | 4.4 ± 11.04* | 6.7 ± 12.83* | 0.313 (k) |
| Mental component^2^ |  |  |  |  |  |
| Baseline | 64.3 ± 10.83 | 64.6 ± 12.94 | 66.6 ± 13.22 | 65.9 ± 11.98 | 0.928 (k) |
| Week 24 | 67.6 ± 14.56 | 68.7 ± 13.40 | 67.5 ± 15.56 | 70.4 ± 12.48 | 0.875 (k) |
| change | 3.3 ± 10.99 | 4.1 ± 11.47* | 0.9 ± 11.29 | 4.5 ± 13.49* | 0.560 (a) |
| Statistical methods: (a) = one-way ANOVA, (k) = Kruskal-Wallis test   1. Physical component includes physical function, physical role activity function, bodily pain, and general health. 2. Mental component includes mental health, social activity function, emotional role activity function, and vitality.   *Statistical significance  *EC* essence of chicken, *CH* collagen hydrolysate, *SD* standard deviation, *SF-36* 36-Item Short Form Survey | | | | | |

**Supplementary Table 4** Food questionnaire where participants were asked how often they ate food in various categories.

| **Food category** | Never | 1 unit per week | 2–3 units per week | 4–6 units per week | One unit per day | > 2 units per day | **1 unit** |
| --- | --- | --- | --- | --- | --- | --- | --- |
| **Dairy**  Milk, milk powder, cheese, yogurt, yogurt drinks, ice cream, etc. | **0** | **1** | **2** | **3** | **4** | **5** | A cup of milk/yogurt drinks (250 mL)  3 teaspoons of dried milk  One yogurt (100 g)  One scoop of ice cream  Two slices of cheese |
| **Nuts**  Sesame, almonds, etc. | **0** | **1** | **2** | **3** | **4** | **5** | A flat teaspoon 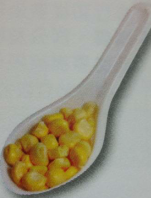 |
| **Meats and protein**  Eggs, soybeans, dried tofu, tofu, soy milk, beef, pork, chicken, fish, dried clove fish, etc. | **0** | **1** | **2** | **3** | **4** | **5** | Soy milk (250 mL)  Meats and protein (half a bowl) 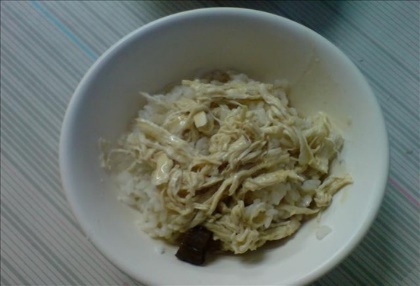 |
| **Vegetables**  Chinese kale, red amaranth, basil, broccoli, etc. | **0** | **1** | **2** | **3** | **4** | **5** | Vegetables (half a bowl) 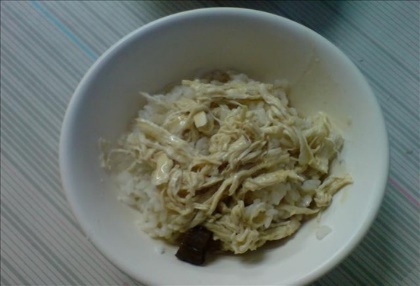 |
| **Mushrooms and dried algae**  Mushrooms, king oyster mushrooms, seaweed, kelp, etc. | **0** | **1** | **2** | **3** | **4** | **5** | Mushrooms and dried algae (half a bowl) 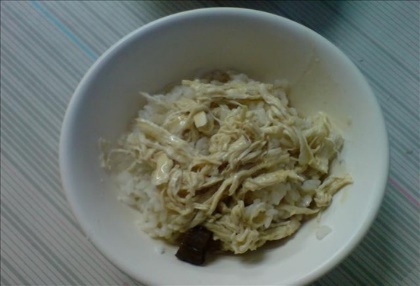 |


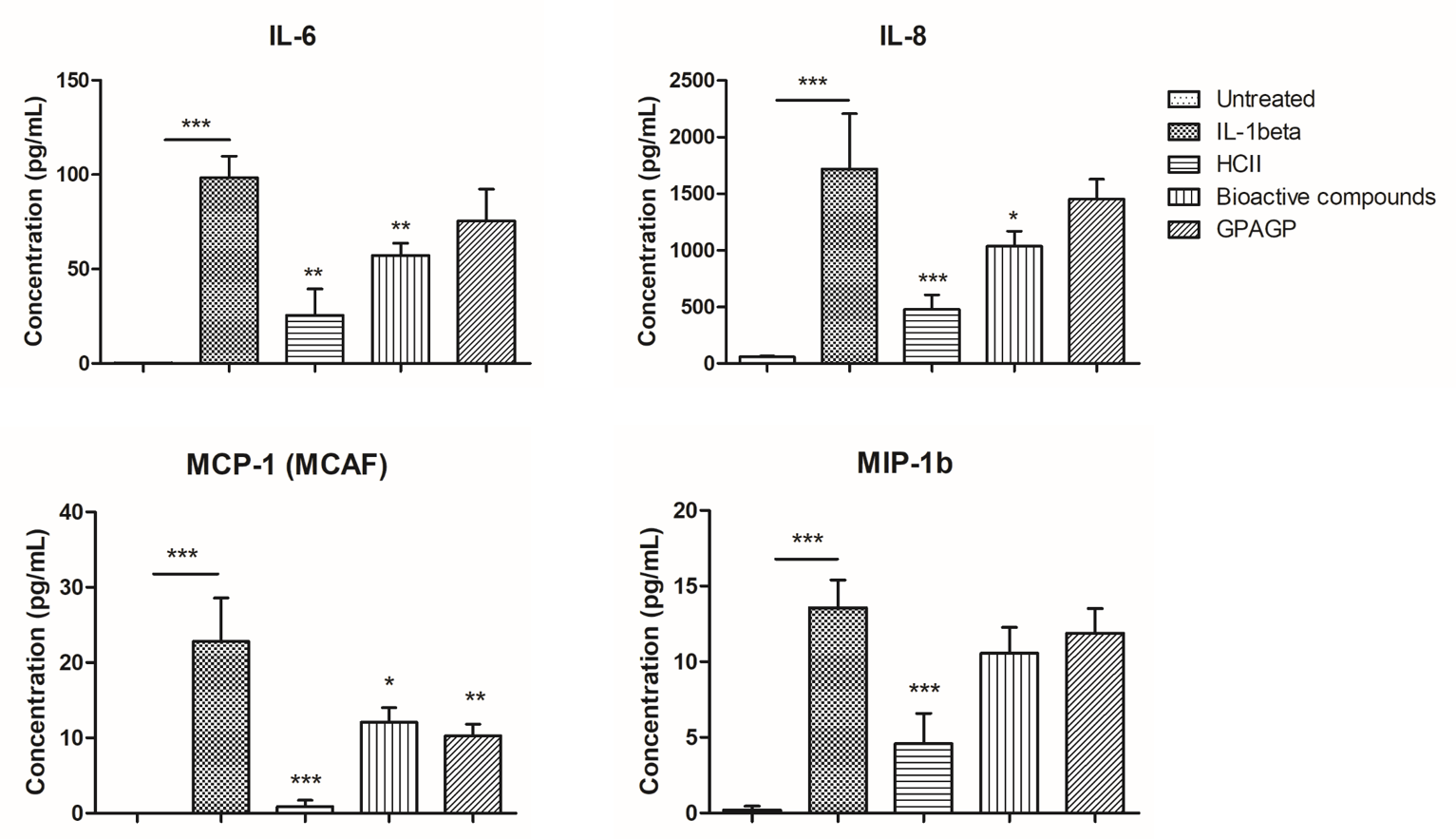


**Supplementary Figure 1. Effects of HC-II and bioactive compounds on the regulation of inflammatory markers.** Inflammation was induced by IL-1β 25 ng/mL and the levels of inflammatory markers (ie, IL-6, IL-8, MCP-1, and MIP-1β) were measured after treatment with HC-II and the combination of bioactive compounds including CPG, CAH, guanosine, and tyrosine as well as the novel peptide GPAGP. Data are represented as mean ± SD (n = 3) against IL-1β unless indicated. *p < 0.05, **p < 0.01, ***p < 0.001
